# Uncertainty of Prenatally Diagnosed Congenital Heart Disease: A Qualitative Study

**DOI:** 10.1001/jamanetworkopen.2020.4082

**Published:** 2020-05-05

**Authors:** Kelly W. Harris, Kathleen M. Brelsford, Ann Kavanaugh-McHugh, Ellen Wright Clayton

**Affiliations:** 1 Division of General Pediatrics, Vanderbilt University Medical Center, Nashville, Tennessee; 2 Center for Biomedical Ethics and Society, Vanderbilt University Medical Center, Nashville, Tennessee; 3 Division of Pediatric Cardiology, Department of Pediatrics, Vanderbilt University Medical Center, Nashville, Tennessee; 4 School of Law, Vanderbilt University, Nashville, Tennessee

## Abstract

**Importance:**

Parents who receive a prenatal diagnosis of congenital heart disease may experience more short- and long-term stress than those who receive a postnatal diagnosis. To identify potential interventions to ameliorate that stress, the longitudinal emotional experience of parents must first be understood.

**Objective:**

To better understand parents’ accounts of their own prenatal experience, particularly aspects they found to be stressful or challenging, and to identify strategies to improve support.

**Design, Setting, and Participants:**

This qualitative study included analysis of transcripts of audio recorded telephone interviews with pregnant mothers and their support persons, as applicable, who were referred to and seen at the Fetal Cardiology Clinic at Vanderbilt Children’s Hospital from May 2019 to August 2019 with an initial likely diagnosis of complex congenital heart disease at any gestational age. Data analysis was conducted from August 2019 to November 2019.

**Main Outcomes and Measures:**

An applied thematic analysis approach was used to code and analyze professionally transcribed interviews. Coding and codebook revisions occurred iteratively; intercoder reliability was assessed and confirmed routinely. One author coded all transcripts; a second author independently reviewed one-fifth of the transcripts at fixed intervals to ensure that interrater reliability remained greater than 80%.

**Results:**

Twenty-seven individuals from 17 families participated in 42 phone interviews during pregnancy, 27 conducted at the first time point after the initial prenatal cardiology consultation and 15 at the second time point after a follow-up prenatal cardiology visit. Most interviewees were mothers (16 interviewees [59%]; median [interquartile range] age, 30.0 [27.3-34.8] years) or fathers (8 interviewees [30%)], with a few support individuals (3 interviewees [11%]) (median [interquartile range] age of family member or support individual, 30.0 [26.0-42.0] years). Initial fetal diagnoses included a range of severe congenital heart disease. Uncertainty was identified as a pervasive central theme and was related both to concrete questions on scheduling, logistics, or next steps, and long-term unknown variables concerning the definitiveness of the diagnosis or overall prognosis. Practitioners helped families through their framing of uncertainty at various time points including before, during, and after the clinic visit.

**Conclusions and Relevance:**

Families walk an uncertain path following a fetal diagnosis of severe congenital heart disease. The challenges faced by the cardiologists caring for them overlap in many ways with those experienced by pediatric palliative care practitioners. Potential future interventions to improve parental support were identified in the areas of expectation setting before the referral visit, communication in clinic, and identity formation after the new diagnosis.

## Key Points

**Question:**

Which aspects of the experience are uniquely stressful for parents receiving a prenatal diagnosis of congenital heart disease?

**Findings:**

In this qualitative study of 27 individuals from 17 families participating in 42 phone interviews, uncertainty—associated with both concrete questions and long-term unknown variables—was a central source of stress for parents.

**Meaning:**

These findings suggest that potential future interventions may focus on parental coping with uncertainty and information delivery by health care practitioners.

## Teaser

This qualitative study used interviews of pregnant women who received a prenatal diagnosis of congenital heart disease to analyze the aspects of the experience that they and their support persons found most stressful and to identify strategies to improve support.

## Introduction

Most diagnoses of congenital heart disease (CHD), which occur in approximately 1% of births in the US, are made prenatally.^1^ Physicians may assume that receiving this diagnosis prenatally is preferred because it allows for additional emotional processing, education, and decision-making about continuing pregnancy and intended delivery location.^2,3,4,5,6,7^ Prenatal diagnosis has been shown to improve parental understanding of CHD at time of neonatal intensive care unit discharge.^8^ Yet evidence suggests that receiving a prenatal CHD diagnosis may be psychologically harmful to parents^9,10,11,12,13^ or provide no psychological benefit.^14,15,16,17,18,19^ Indeed, some parents who receive these diagnoses may experience more stress, anxiety, and depression at hospital discharge and for months after birth.^9,10,11,12,13^

Counseling practices in fetal cardiology overlap in many ways with those of pediatric palliative care, a specialty dedicated to goal setting, improving quality of life, and relieving suffering.^20^ Exploring parallels between the 2 fields may be helpful as fetal cardiologists work to improve parental support and coping with prenatal diagnosis. Indeed, in 1 study,^21^ early palliative care psychologically benefited mothers who had received a prenatal CHD diagnosis and improved family communication and relationships.

To develop effective interventions to support these parents, we must first understand the unique challenges they face, research that others have called for.^18,22,23,24,25,26,27^ Although prior qualitative studies have begun to investigate the experience of mothers after receiving a prenatal diagnosis,^22,28,29,30,31,32,33,34,35,36^ this study extends that knowledge by focusing on those receiving a prenatal CHD diagnosis in the US, including other family members, and tracking parents’ experience over time. The goal of this study is to learn what aspects of prenatal diagnosis are particularly stressful for prospective parents and to identify potential interventions that clinicians may take to ameliorate that stress.

## Methods

### Study Design and Setting

This study was approved by the Vanderbilt University institutional review board. Written, informed consent was obtained from all participants at enrollment in addition to verbal consent before each interview. This study follows the Consolidated Criteria for Reporting Qualitative Research (COREQ) reporting guideline for qualitative studies.

We conducted a qualitative study of pregnant mothers and their partners, spouses, or other support persons who were referred to and seen in the Fetal Cardiology Clinic at Vanderbilt Children’s Hospital for likely complex CHD diagnosed at any gestational age. Complex CHD was defined by categories of diagnosis severity,^37^ either moderate or high risk of affecting life expectancy, or complexity greater than or equal to 3 on a prognosis scale.^38^ Scores of 3 to 6 include lesions that can be surgically repaired to nearly normal anatomy, and scores of 7 to 10 encompass single ventricle repairs (ie, scores that are roughly equivalent to Risk Adjustment for Congenital Heart Surgery risk categories 2 to 5, which stratify the risk of cardiac surgery).^39^ Diagnoses were not confirmed at the time of enrollment, so a few families with diagnoses of lower complexity at birth were included. Non-English speakers and those who speak English as a second language were included when they requested counseling in English or an in-person interpreter was present for parental counseling in clinic. Race/ethnicity was defined by the participant either in person or in their medical record.

Individuals were enrolled before the pregnant mother’s first consultation appointment in fetal cardiology. As part of the larger study, which includes postnatal interviews of participants, in-clinic prenatal counseling was observed at the initial prenatal cardiology appointment and 1 follow-up visit. All families were counseled by 1 of 4 participating fetal cardiologists who had consented to participate in the study.

The same investigator (K.W.H.) sought to conduct semistructured interviews either in person or over the phone with each individual participant at 3 time points, 1 after each observed prenatal visit and 1 postnatally. She audio recorded all interviews with permission.

### Data Collection

Participants were enrolled from May 2019 to August 2019; 31 families were approached on the basis of the stated reason for their referral by the referring physician. Of those approached, 5 families declined enrollment, and 5 other families enrolled but subsequently were excluded after normal echocardiograms. Of the 37 individuals from 21 families who were enrolled and qualified for the study, 10 individuals were lost to follow-up before any interviews were conducted. Active participants included 27 individuals from 17 families, leading to 42 interviews conducted prenatally at time points 1 and 2 from May 2019 to October 2019 (Figure). Time point 1 occurred after the initial prenatal cardiology consultation at gestational ages of 20 to 35 weeks (median, 25 weeks). Time point 2 occurred after a follow-up prenatal cardiology visit at gestation ages of 29 to 37 weeks (median, 32 weeks).

**Figure.**
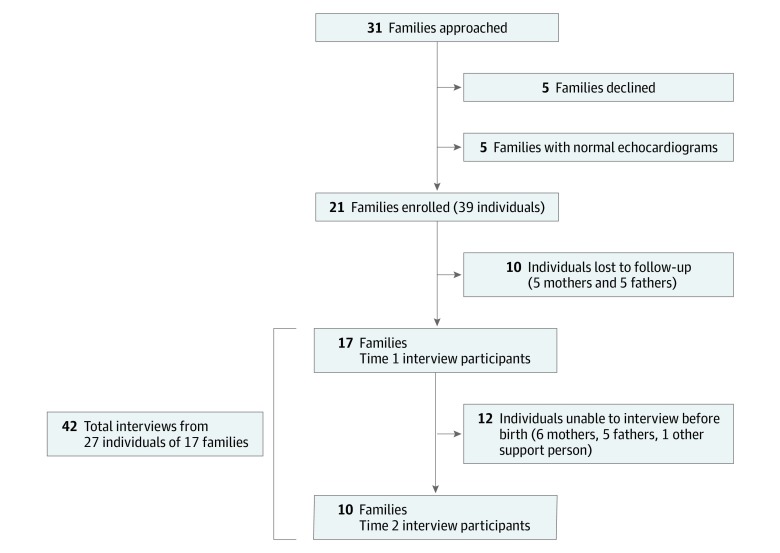
Study Cohort Flowchart, Including Initial Participation in Clinic and Participation in Semistructured Phone Interviews Over Time

A qualitative, semistructured interview guide with 13 primary questions was used to explore a participant’s overall story, anticipation of the future, factors influencing their experience, and feelings of empowerment across 3 time points (see the eAppendix in the Supplement). This article reports their responses to questions about their experience before the clinic visit, feedback about the clinic visit, and reflections on current understanding, as well as fears, hopes, and dreams. The mean interview length was 29 minutes. Participants were mailed $20 gift cards after each interview as a token of appreciation for their time. Audio recordings were professionally transcribed verbatim with personal identifiers removed. Before deidentification, additional fetal and obstetrical information was obtained from the mother’s electronic health record.

### Statistical Analysis

 Data analysis was conducted from August 2019 to November 2019. Transcripts from interviews at both the first and second time points were uploaded to NVivo data analysis software version 12 (QSR International) and were analyzed as 1 group. An iterative process was used to code and analyze data using an applied thematic analysis approach.^40^ One author (K.W.H.) developed an initial structural and content codebook including code definitions, inclusion and exclusion criteria, and examples.^41^ Two authors (K.W.H. and K.M.B.) then met to refine the codebook through application to 1 transcript. They then independently coded 3 transcripts to reach intercoder reliability of at least 80%. K.W.H. coded all transcripts; K.M.B. independently reviewed one-fifth of remaining transcripts at fixed intervals to ensure that interrater reliability remained greater than 80%. Any discrepancies in coding were resolved through discussion until a consensus was reached.

In this article, participant quotations are identified first by family number (1-21), then by participant role (M = mother, F = father, O = any other support person as shown in Table 1), and finally by time point of interview (1-2). Thus, 5.F.2 indicates a quotation from a father of family number 5 during the second interview.

**Table 1.** Demographic Characteristics of Participants Who Received a Prenatal Diagnosis of Congenital Heart Disease

Characteristic	Participants, No. (%)
Individual information	
Role	
Mother	16 (59)
Father	8 (30)
Grandmother	1 (4)
Great-grandmother	1 (4)
Friend	1 (4)
Race/ethnicity	
White (European)	20 (74)
White (Middle Eastern)	1 (4)
Black or African American	3 (11)
Hispanic or Latino	3 (11)
Age, median (interquartile range), y	
Mother	30.0 (27.3-34.8)
Family member or support person^a^	30.0 (26.0-42.0)
Occupation	
Medical field^b^	9 (33)
Business or sales	8 (30)
Homemaker	6 (22)
Mechanic, electrician, or construction	2 (7)
Lawyer	1 (4)
Firefighter	1 (4)
Total	27 (100)
Family information	
Distance of mother from hospital, median (interquartile range), miles	32.0 (14.5-92.0)
Gestation at first consultation visit, median (interquartile range), wk	25 (21-32)
Primary language	
English	14 (82)
Spanish	1 (6)
Arabic	1 (6)
Other (African dialect)	1 (6)
Gravidity of mother	
1	3 (18)
2	6 (35)
3	1 (6)
4	4 (24)
5	2 (12)
7	1 (6)
Parity of mother	
0	3 (18)
1	6 (35)
2	5 (29)
3	2 (12)
4	1 (6)
Prior miscarriages	
0	12 (71)
1	3 (18)
2	2 (12)
Family history of congenital heart disease	
No	12 (71)
Yes	5 (29)
Fetal diagnosis	
Pulmonary stenosis or atresia	3 (18)
Coarctation of the aorta	3 (18)
Tetralogy of Fallot	2 (12)
Ebstein anomaly	2 (12)
Transposition of the great arteries	2 (12)
Hypoplastic left heart syndrome	2 (12)
Hypoplastic right heart syndrome	1 (6)
Dilation of the aorta	1 (6)
Atrioventricular septal defect	1 (6)
Current baby vital status post partum	
Alive	12 (76)
Deceased	5 (24)
Total	17 (100)

SI conversion factor: To convert miles to kilometers, multiply by 1.6.

^a^ Three data points are missing because the ages of 2 fathers and 1 great-grandmother are unknown.

^b^ Includes caregiver, health care administrator, health care coder, health systems worker, home health assistant, nurse, physiologist, and imaging technician.

## Results

### Demographic Characteristics

Demographic characteristics are listed in Table 1. Of the 42 recorded phone interviews conducted prenatally, most were with mothers (16 participants [59%]; median [interquartile range] age, 30.0 [27.3-34.8] years) or fathers (8 participants [30%]), and a few with support individuals (3 participants [11%]) (median [interquartile range] age of family member or support individual, 30.0 [26.0-42.0] years). The majority self-identified as white (21 participants [78%]). The primary language of most families was English (14 families [82%]); only 1 family use a language interpreter for counseling or the interview. Most mothers had other live children (14 mothers [82%]) and no known family history of CHD (12 mothers [71%]). Five mothers (30%) had experienced a prior miscarriage. Initial diagnoses included a variety of cardiac anomalies.

### Prenatal Uncertainties

After extensive counseling in fetal cardiology clinic, participants commonly expressed remaining uncertainty in 2 areas: concrete logistical questions with knowable answers and more long-term unknown variables that simply required acceptance. A thematic analysis summary of illustrative quotations from some participants is shown in Table 2.

**Table 2.** Thematic Analysis Summary of Illustrative Quotations From Participants on Their Feelings of Uncertainty

Theme	Illustrative quotations^a^
Concrete questions	“Them just explaining everything to us and how it’s going to go. The surgery, what they’re going to do to the heart. … So just the more information really helps you grasp what is going to happen. And the timeframe, too. After the first appointment we were kind of still like what's the timeframe like?” (2.M.2)
	“I guess I don’t like, I don’t know a lot of the, I guess the process now. … I have genetics, I mean, you know, just all kinds of different appointments and like, oh I don’t really know. Do I still go see my typical OB that I was gonna see cause the ones throughout the pregnancy or am I strictly gonna see doctors for high risk pregnancy? I don’t really know that.” (5.M.1)
Long-term uncertainties	“And then she said we don’t know until we see him. Nothing’s inevitable. I’m not going to plan for anything then because, that makes sense. You don’t know what you're doing until you open him up and see it. Until his scan versus going through me to scan.” (5.M.2)
	“It’s kind of tough being in the dark, but you know in the situation you can’t really do too much about being in the dark just because it changes, and evolves from day to day, or month to month.” (5.F.2)
	“Well, there’s the unknown is that even [the fetal cardiologist] couldn’t give a clear answer and you just have to accept that for now. It would be ideal if she said, ‘Definitely this is going to happen or not happen.’ And so that's what everyone keeps asking me like, ‘Are you okay with it?’ I'm like, ‘Well, I just wish I knew what to expect as far as are we going to be in the hospital for a few weeks or is she going to come home?’ Those types of things but no one can answer those until they look at her and check her out. I understand that part, but it’s still hard because usually you say someone’s sick and they get immediate treatment and here we are just waiting to get the treatment that she will need.” (8.M.2)
	“Yeah, I think for us, the question has been mostly we wish that we could have a 100% definitive diagnosis, which is super challenging … That’s hard to do, diagnosing in utero, anyways. It’s amazing what they can tell. But for us that would give us a lot more peace of mind. And that’s definitely not medical-side’s fault, it’s just [inaudible] versus what we can see and confirm through fetal echoes. But I think that’s been one of our main questions. And then just some of the unknowns that nobody can predict, even in a normal healthy delivery, how baby’s going to respond.” (12.M.1)
Impact of practitioners on family perceptions and narratives	
Before the appointment: expectation setting	“Well we were doing a regular ultrasound, and the doctor found something that she really didn’t like, so she sent us to a different doctor. And that doctor told us that, what she told us was different than what you guys told us. … And I mean we got there thinking it was one thing, and y’all made it clear for us that it was a completely different thing, and that it wasn’t as scary as she made it out to be.” (13.M.1)
	“When we got there the ultrasound, it took little pictures of the baby, and through all those pictures, I think the doctor told us that the baby had a hole in his heart. So, that gave us a lot of stress. Because, we didn’t know how to process that, what it meant for them to say that the baby had a hole in his heart.” (13.F.1)
	“First of all, I was like, well what’s going on? I mean, what is not normal, is there something really bad or what? And they say they don’t really know … they cannot tell me anything else because they don't really know what it was. They say, we just want you to see the cardiology and they going to tell me what it is.” (15.27.1)
During the clinic visit: nonverbal communication	“I think that was harder the first appointment, that was hard because everyone was so happy and upbeat and talkative, because no one’s making frowning faces at the computer and sad for me already. So that’s when I was blindsided that things were bad in the consultation afterwards. But this time the ultrasound’s basically the same, everyone’s still basically happy. And so I felt like, I kept thinking back. I was like, all right, well every one’s decently happy in the first one too, and it was still bad, so I just prepared myself for the consult, which is fine because they do it all day, if they just sat around and it was constantly sad, I imagine their life would be awful. So, it’s not like I need the nurse doing my ultrasound, was so caring, to have a frowning face and be quiet and somber the whole time. But, I guess that’s completely irrational and unreasonable to expect of someone. But it’s still a little misleading.” (5.M.2)
	“I was just thinking that, I don't think my baby is okay because since’ I’ve been here like an hour, this thing taking a lot of pictures. And this thing requesting for pictures, I was just starting to think myself that I don't think everything is okay with my baby.” (6.M.1)
After the clinic visit: identity formation	“Everybody is different so just because someone’s heart or body is slightly different doesn’t necessarily mean it’s defective. And that was really eye opening to me. And honestly just that simple explanation of the reason they choose their words. It took a lot of stress off of me… when they said that to me, you know, that just because her heart was this way, that it’s different, doesn’t necessarily mean that it’s defective. I mean it, really like, I could feel myself breathe better” (4.M.1)
	“Probably all of it. I think my most probably best thing that I got was the support groups. … I'm like not the only person in the entire world that's going through this. And I know that, but like it’s nice to kind of hear that, and that to me was probably the most impactful thing. Yeah, so that was good.” (11.M.1)

^a^ Participant quotations are identified first by family number (1-21), then by participant role (M = mother, F = father, O = any other support person as shown in Table 1), and finally by time point of interview (1-2). Thus, 5.F.2 indicates a quotation from a father of family number 5 during the second interview.

#### Concrete Questions

Receiving information about appointments and scheduling, logistics, and future steps was valuable to participants in addressing some uncertainty and allowing participants to feel some sense of control or awareness of the process. One mother thought understanding “the process of what we were going to be expecting…kind of relieved a lot of unknown because we had no clue what was going to happen…other than our baby’s heart was messed up” (2.M.1). Parents expressed confusion and uncertainty regarding a range of processes, from “donating organs” (10.F.2) to scheduling “an MRI” (20.O.1), from “Who’s calling me to set stuff up?” (8.F.1) to whether “I still go see my typical OB … or am I strictly gonna see doctors for high risk pregnancy?” (5.M.1).

More than one-third wanted more communication about “an appointment plan” (8.F.1) and “the plan of action, and the coordination of care” (8.M.1). One father was frustrated that “no one contacted me after we did the ultrasound or the visit” (7.F.1). One native Spanish–speaking mother, who had refused an interpreter in clinic, expressed global uncertainty saying, “I’m still a little confused… when you don’t speak English very well and you misunderstand some things” (15.M.1). To handle these addressable, concrete challenges, 1 mother recommended “an advocate...they could probably communicate a lot faster and get an answer faster than all the parents calling [the office] and bombarding them with their caseload” (8.M.1).

#### Long-term Uncertainties

Prenatal counseling for CHD also involves unavoidable uncertainties for both parents and practitioners. Counseling in these cases included the practitioners’ assessment of the anticipated severity of the lesion and prognosis as well as any uncertainties in the diagnosis, the known limitations of fetal echocardiography, and the need for confirmation of the anatomy postnatally. Uncertainties for families included lack of a “100% definitive diagnosis” and “some of the unknowns that nobody can predict, even in a normal healthy delivery” (12.M.1).

All families hoped for “a perfect healthy kid with no complications … [who] is able to do whatever his heart desires” (5.F.1), and one-third wished that “we could predict the future” (2.M.2), yet everyone recognized that even the best physicians simply could not provide them with certain information regarding every aspect of diagnostic severity and prognosis before delivery. As 1 father explained, “We realize that’s a gray area until that day comes where we can get a better scan on his heart” (11.F.1). Although families often accepted the inevitability of uncertainty and hoped for “the best-case scenario” (8.F.1, 12.M.2, and 13.M.1), the “unknown of it all” was nonetheless a source of great fear (3.M.1).

The need to wait before definitive diagnosis and intervention loomed large. Parents were frustrated about uncertainty in “the near future and the distant future” (4.M.1), from “not knowing what to expect in the next few weeks” (8.M.1) to uncertainty about whether they would be “bringing a child home from the hospital” (12.M.1) and whether the child would “have multiple surgeries or a transplant” (1.O.2). Individuals worried both about “losing the baby” (10.M.1) and that the baby would survive but have “a lifelong [sic] of medical problems and have a terrible quality of life” (5.M.1). Approximately one-fourth believed they would feel a sense of certainty at birth, but approximately one-half feared the heart abnormality would haunt them throughout their child’s life.

Approximately one-fourth of participants expressed uncertainty regarding their ability to adequately prepare for life as parent, particularly of a child with a heart difference. Fathers often expressed concern about their ability to respond to expected future “financial difficulties” related to health care costs associated with a possible heart condition (8.F.2). In contrast, only women worried about their ability to fulfill their familial roles as mothers or grandmothers. One mother feared that “as a mother I won’t be able do everything that I need to do” (4.M.1). Even delivery can feel threatening; one said, “for now the baby’s good … but, knowing that when the cord is cut, she’s on her own, so that sounds like a big fear right now” (12.M.2).

### Impact of Practitioners on Family Perceptions and Narratives

The ways in which practitioners framed uncertainty affected participants’ own perceptions, experiences, and narratives. Participants understandably felt “heavy-hearted” (5.F.1) during counseling. The strategies used by practitioners before, during, and after appointments were perceived by families to reduce or compound stress surrounding uncertainty.

#### Before the Appointment: Expectation Setting

Referring physicians find themselves in a challenging position: providing parents with too much information regarding an unconfirmed diagnosis may cause parents undue anxiety, but providing too little information about the reason for the referral could also create concern. At least 6 families (35%) were confused about the potential diagnosis for which they were being referred. “[The obstetricians] were just saying that there was something that we are not very certain with regards to the heart” (7.F.1).

Families interpreted referring physicians’ explanations in a range of ways, from thoughtful to deceitful. Some (4 participants [15%]) believed only limited details were disclosed “because [the obstetricians] didn’t want to encourage me or discourage me without knowing” the exact diagnosis (5.M.1). Others (4 participants [15%]) thought the lack of information was “not very kind” (8.M.1) or indicated “something really bad” about the diagnosis (15.M.1). One father said that the vague diagnosis from the referring physician “gave us a lot of stress, because we didn’t know how to process that, what it meant” (13.F.1). For him and other participants, knowing more about the potential diagnosis, even if it was severe, would have been less stress inducing. Another set of families arrived at clinic thinking they knew the diagnosis and were told “a completely different thing” that “wasn’t as scary as [the obstetrician] made it out to be” (13.M.1). Even when the diagnosis was consistent, practitioners who communicated “differing information” about the prognosis (4.M.1) caused “a lot of ups and downs” (20.M.1).

#### During the Clinic Visit: Nonverbal Communication

Participants had divergent perceptions about what was going on during a clinic visit. Many looked to every practitioner, including the sonographer, for clues on the prognosis. For example, when everyone in the ultrasonography room is “upbeat the whole time” parents interpreted the mood to indicate a positive prognosis (5.M.2). Three participants described being “blindsided because I had no clue during the scan” about bad news (12.M.1). Others concluded from extended ultrasonography scans or prolonged delays that “there’s something they’re not telling us” (8.F.2) or “I don’t think my baby is okay” (6.M.1).

#### After the Clinic Visit: Identity Formation

After the clinic visit, participants formed a new family identity incorporating the prenatal diagnosis. They often discussed how the words practitioners used helped to shape that narrative. As an example, many parents blamed themselves for the CHD diagnosis, but they learned from practitioners that neglect or a mistake was not the cause for the abnormal heart formation. One mother said the most important thing she learned in clinic was “I wasn’t doing something wrong…things happen” (15.M.1).

Some families noted that the specific words used to describe a heart condition had powerful effects on their own perspective. Two mothers described the positive impact of intentional language used by their fetal cardiologist. One explained, “I vividly remember…it feeling almost offensive to say, your daughter has a heart defect…it was just so sweet, everyone referring to it as a heart difference. Because hey, everybody’s body doesn’t look the same in a lot of different ways” (12.M.1). Another said, “My doctors have been really great, especially the cardiology clinic, calling her heart things a difference and not a defect. I think just the words that the doctors choose makes a huge difference” (4.M.1). Later in the interview, she explained that those words taught her that “different doesn’t necessarily mean…defective” (4.M.1). Hearing the new framing “took a lot of stress off…I could feel myself breathe better” (4.M.1).

Certain practitioner communication strategies helped families reframe the diagnosis to see that each individual has a unique story, regardless of his or her prognosis. Participants described how the diagnosis left them “heartbroken” (6.M.1) and shifted their attention away from joyful future plans like “putting a nursery together” (5.M.2), “wearing dresses and hair bows” (20.O.1), or “playing sports” (1.M.1). Yet 1 father realized, “[If] having a heart condition definitely limits his ability to participate…he could still just enjoy being a fan or learning about the sports” (5.F.1). Another said, “there’s plenty of jobs and plenty of things that you can do that don’t require you to be physically [able]” (10.F.1). These parents redefined what they were hoping for.

One-quarter of participants felt that connecting with other parents was helpful. Some simply appreciated having support group information and “tools to reach out if I needed” (2.M.1). Engaging with other families reminded them that “I’m not the only person in the entire world that’s going through this” (11.M.1). Others benefited from “just hearing different stories” (1.M.2) from families who had previously received a CHD diagnosis prenatally. Some thought those connections could be useful “to prepare for us coming” to the clinic (18.F.1), to know where the parking lot or cafeteria are located, or “the quickest way to get to the fifth floor” (10.F.2). On the other hand, 5 participants thought it would be harmful to talk with another family because “every child is completely different” (18.F.1), so it might cause “false hope” (10.F.2) or “doubt” and “worrying” when “their experience is completely different…sometimes ignorance is bliss” (5.F.2).

## Discussion

The development and incorporation into routine use of prenatal diagnostic tools enables prospective parents and their health care practitioners to learn more about the unborn child.^42,43^ Perhaps the most powerful of these are technologies such as ultrasonography that permit the fetus to be visualized. Because most pregnancies are uneventful, these scans usually serve as a source of reassurance and often promote parent-child bonding.^44,45^

The detection of abnormalities may offer the possibility of intervention to improve fetal outcomes, as in the case of fetal surgery for spina bifida.^46,47,48^ Most of the time, however, problems identified in unborn children cannot be ameliorated or even fully defined during pregnancy. In these cases, prospective parents are left in a new and different “expectant” or liminal state. Although they may grieve the healthy child for whom they had hoped,^49,50^ they face an extended period during which they can only try to plan for the future with and for a child who may have serious medical problems but whom they have not yet held.^38^

The prenatal diagnosis of serious CHD poses particular challenges for families. For the past 35 years, fetal cardiac screening has been a routine part of the prenatal 20-week anatomy ultrasonography.^42^ Often, the exact diagnosis and specific plan of treatment cannot be determined until after the child is born, but frequently, the prospect of major surgery shortly after delivery looms. Meanwhile, parents can face concrete challenges in making clinic visits and planning where to deliver their child. Not surprisingly, a diagnosis of CHD causes parental stress from the time of diagnosis through cardiac surgeries and afterward,^51^ often causing them more anxiety, depression, and posttraumatic stress than other parents experience from having children with other medical conditions.^51,52,53,54,55^ Parental stress, in turn, is associated with decreased physical and psychological well-being in both children and their parents.^56,57,58^ Indeed, parental stress can have greater effect on quality of life for children with CHD than their illness severity.^59,60,61,62^

Consistent with Merle Mishel’s uncertainty in illness theory,^63,64^ when faced with uncertainty from a prenatal diagnosis of CHD, parents searched for information to reduce the uncertainty as well as for strategies to cope with it. Previous research has reported on the importance of information gathering in parental coping after a prenatal CHD diagnosis.^31,33,34,36,65,66,67^ Health care professionals can help parents by more explicitly framing the information they provide.^68^

Fetal cardiologists regard parental counseling as integral to any encounter and are committed to improving it in their clinics.^27^ The Fetal and Perinatal Learning Laboratory within the National Pediatric Cardiology Quality Improvement Collaborative allows cardiologists in the United States to work to optimize communication and support for families receiving prenatal CHD diagnoses.^69^ Thus, this group is attempting to standardize the information provided across institutions regarding hypoplastic left heart syndrome by providing checklists of recommended discussion topics to patients and practitioners.

In this study, families identified many aspects of counseling from fetal cardiologists they valued, including verbal and written communication in lay language about the cardiac diagnosis, resources for ongoing support, and general patience and kindness. They expressed gratitude to all practitioners in this clinic for establishing clear expectations, including reason for referral, definitions of practitioner roles, outline of clinic visits, and next steps, including a precise timeline. They also appreciated how practitioners addressed common fears, selected word choice carefully, and described the possible positive future abilities of their unborn child. All fetal cardiology team members built trust with parents by addressing these concrete uncertainties and closing the gap in communication.^34,70,71,72^

We suggest that in these ways fetal cardiologists are implementing practices that overlap with those used by pediatric palliative care to address parental concerns. Future interventions to improve parental support may focus on honing these already well-developed skills. For example, as suggested by 1 study participant, communication may be further optimized through integration of a patient navigator who can check in more frequently with families and help to translate the logistics of the medical world, as has been successfully implemented in other areas of medicine.^73,74^ Communication training activities would give feedback to practitioners on intended vs perceived messaging. Role-playing activities could elucidate specific language and sensitive framing that are less alienating.^75^ For example, robust literature has supported the medical field’s shift in terminology from *mental retardation* to *developmental delay* or *intellectual disability*.^76^ Similarly, future studies of parents with children diagnosed with CHD may recommend using the term *heart difference* over *heart defect*, as was done by practitioners in this study.

Palliative care practice hinges on understanding an individual’s hopes, dreams, and fears within their social context.^77^ Grasping those aspects of an individual’s story creates a window to understanding how their present health care experience fits into their personal narrative. In the interview guide, those topics were intentionally explored with each study participant, many of whom volunteered that the interview itself was helpful and therapeutic. Fetal cardiologists may engage intentionally in such conversations, gaining trust and helping parents redefine what they hope for their child.^28^ However, given the already extensive counseling performed at fetal cardiology clinic visits, cardiologists may instead choose to engage their pediatric palliative care colleagues to explore those topics with parents. Teams may also learn the limits of acceptable interventions for a family, which would help inform future neonatal intensive care unit conversations.

In this study, participants seemed to value most highly an explicit plan for what happens today, throughout the remainder of pregnancy, immediately after delivery, and in the more distant future. Sometimes practitioners may hesitate when asked for such plans given the number of unknown variables inherent to prenatal CHD diagnosis. However, family members are not necessarily asking for certainty, but instead for information about the possible pathways that may be taken, understanding that the choice of path may be shaped by the child’s condition. As Asplin et al found, mothers ask “for more and explicit information both verbal and written including best- and worst-case scenarios.”^67^ Participants in the present study defined their hopes and fears by what they imagined those best-case and worst-case scenarios to be, and they referenced in subsequent interviews how new medical information helped them understand which path they seemed to be on, influencing their vision for the future. This approach to facing and dealing with uncertainty is also common among patients receiving palliative care.^78^

### Limitations

This study has limitations that should be taken into consideration. The purpose of the study was to describe families’ experiences between diagnosis and delivery. Future studies conducted at multiple institutions with a greater diversity of families and experience after delivery can further inform knowledge on this important topic. Most mothers interviewed in this study had already had a successful pregnancy. The experience of women who do not have prior children may be different, with distinct needs. At the same time, 2 of the families in this study had prior children with complex CHD and so had unique perspectives. Future research should investigate how these attributes affect informational needs.

## Conclusions

Irresolvable uncertainty following prenatal diagnosis of CHD is a primary source of stress for parents. Fetal cardiologists support parents in many ways that overlap with pediatric palliative care practices: addressing answerable questions, framing the parental experience within a larger context, and walking alongside families in the unknown. Family members value fetal cardiology practitioners discussing the plan, setting more supportive framing, and acknowledging the existential nature of some information that leads to a range of emotional reactions and coping strategies. Future studies should investigate the effectiveness of interventions on clarifying uncertainties or improving parental coping with uncertainty.

## Notes

Supplement. **eAppendix.** Interview Guide
