# Supplementary material for: Uncertainty of Prenatally Diagnosed Congenital Heart Disease: A Qualitative Study
Source: JAMA Netw Open. 2020 May 5;3(5):e204082. doi: 10.1001/jamanetworkopen.2020.4082 (PMC7201310; doi:10.1001/jamanetworkopen.2020.4082)
Supplement: Supplement. — eAppendix. Interview Guide [file jamanetwopen-3-e204082-s001.pdf]

Supplementary Online Content

Harris KW, Brelsford KM, Kavanaugh-McHugh A, Clayton EW. Uncertainty of prenatally diagnosed congenital heart disease: a qualitative study. *JAMA Netw Open*. 2020;3(5):e204082. doi:10.1001/jamanetworkopen.2020.4082

eAppendix. Interview Guide

This supplementary material has been provided by the authors to give readers additional information about their work.

© 2020 Harris KW et al. *JAMA Network Open.*

eAppendix. Interview Guide

Narrative and understanding:

- I want to hear your story.
  - First interview: When did you first find out that your baby could have a heart problem? How did you get to the Fetal Cardiology Clinic?
  - Second interview: I want to hear your story since your first visit to the Fetal Cardiology Clinic. What has happened?
- What did you learn from your appointment?
  - Can you describe what is going on with your baby? What is your understanding of your baby's diagnosis and how it might affect his life and your family's life?
  - Can you describe the options you have in your own words?
  - Do you have questions about what is going on?
  - Are there things you wish you knew or did not know?

Role of the providers:

- What was your experience at the Fetal Cardiology Clinic?
  - How did you feel during the ultrasound? During counseling?
  - Did you feel like you understood what was going on?
  - How do you feel about the amount of information you were given?
    - What was important?
    - Are there things you wish had been communicated differently?
  - Some parents say that when they hear a diagnosis, they then go blank and hear nothing else. Others say that they become extra focused and remember every word. How did you respond to hearing the diagnosis?
- What do you remember most about the providers in Fetal Cardiac Clinic?
  - Was there anything that stuck out to you?
  - Is there anything that could have gone differently?

Anticipation:

- What is the next step?
  - How will you prepare?
  - What will be your role?
  - What does this problem mean to and for you and your family?
- You are going through a clearly stressful time in life.
  - What is your greatest fear? What are you most worried about?
  - What are you and your family hoping for?
  - What do you imagine for your child?

Influencing factors:

- On a 1 to 10 scale, how much control do you feel like you have? Why?
- On a 1 to 10 scale, how stressed are you currently? Why?
  - What are some of the other things going on in your life?
  - How has the stress been affecting your family?
    - Do you have any other children?
    - Do you care for anyone else?
  - How is this affecting your job?
  - Is there anything in your personal medical history or in your family's medical history that has prepared you in any way for this experience?
- On a 1 to 10 scale, how well do you think you are coping?
  - How are you coping?
  - How do you typically respond to stress or difficult times?
  - How are you now coming to accept this diagnosis and move forward in life?
- On a 1 to 10 scale, how supported do you feel?

© 2020 Harris KW et al. *JAMA Network Open.*

        - Where are you getting support from?
        - Family? Friends? Internet? Church?
        - Have your relationships been impacted during this pregnancy?

Resilience and empowerment:

- How difficult has this experience been for you? For your family?
    - Does anything make it better?
    - Can you think of anything your provider could do to make it better?
        - Identify another parent to talk with?
        - Give you a letter that details your child's condition simply?
        - Provide a list of reputable websites?
        - Identify a specific support group?
- Imagine a parent like you was referred to Fetal Cardiac Clinic for evaluation of a similar diagnosis:
    - What would you tell them to expect?
    - What questions would you suggest they ask?
    - What would you want the physician to make sure to say or do?

© 2020 Harris KW et al. *JAMA Network Open.*
